# A chromosome-level genome assembly of the Korean crossbred pig Nanchukmacdon (*Sus scrofa*)

**DOI:** 10.1038/s41597-023-02661-7

**Published:** 2023-11-03

**Authors:** Daehong Kwon, Nayoung Park, Suyeon Wy, Daehwan Lee, Han-Ha Chai, In-Cheol Cho, Jongin Lee, Kisang Kwon, Heesun Kim, Youngbeen Moon, Juyeon Kim, Woncheoul Park, Jaebum Kim

**Affiliations:** 1 Department of Biomedical Science and Engineering, Konkuk University, Seoul, 05029 Republic of Korea; 2 Animal Genomics and Bioinformatics Division, National Institute of Animal Science, RDA, Wanju, 55365 Republic of Korea; 3 Subtropical Livestock Research Institute, National Institute of Animal Science, RDA, Jeju, 63242 Republic of Korea

**Keywords:** Comparative genomics, Genome assembly algorithms

## Abstract

As plentiful high-quality genome assemblies have been accumulated, reference-guided genome assembly can be a good approach to reconstruct a high-quality assembly. Here, we present a chromosome-level genome assembly of the Korean crossbred pig called Nanchukmacdon (the NCMD assembly) using the reference-guided assembly approach with short and long reads. The NCMD assembly contains 20 chromosome-level scaffolds with a total size of 2.38 Gbp (N50: 138.77 Mbp). Its BUSCO score is 93.1%, which is comparable to the pig reference assembly, and a total of 20,588 protein-coding genes, 8,651 non-coding genes, and 996.14 Mbp of repetitive elements are annotated. The NCMD assembly was also used to close many gaps in the pig reference assembly. This NCMD assembly and annotation provide foundational resources for the genomic analyses of pig and related species.

## Background & Summary

Recent advances in whole genome sequencing technology have made it possible to obtain large amounts of genome-wide information at a relatively cheap cost in a short time. Based on the relatively short sequencing reads, several genome assembly algorithms have been proposed to reconstruct whole genomes of target species^1^. However, repeat elements scattered in the genomes make it difficult to fully reconstruct the genomes at intact chromosome level and rather generate short fragmented sequences. Diverse additional data providing long-range genomic information have been used to join fragmented sequences and extend them into chromosome-level assemblies^2^. Recent studies have successfully reconstructed chromosome-level assemblies using Hi-C sequencing reads^3–5^ and optical maps^6,7^. Although these are practical resources, they are still prone to produce mis-assemblies because of their noise and low resolution. In addition, these methods require deep coverages thus large amounts of resources^8,9^.

When high-quality genome assemblies of related species are available, a reference-guided approach can be a good alternative solution for high-quality assembly reconstruction^10^. The approach joins contigs and scaffolds using the additional information obtained from orthologs among target and reference species. This is cost-effective approach for building high-quality assemblies, even at the chromosome-level, without requiring additional sequencing costs. Several recent studies have successfully built chromosome-level assemblies of diverse species based on the reference-guided approach and revealed unique genomic features of the species by comparing the assemblies with those of different related species^11,12^. These studies have also demonstrated the impacts of those genomic features on phenotypes and underlying biological mechanisms by performing multi-omics analyses together.

We applied the reference-guided approach to construct a genome assembly of the Korean crossbred pig, which is called Nanchukmacdon, derived by mating three different commercial breeds (Korean native pig, Duroc and Landrace) in Korea^13^. Nanchukmacdon shows improved meat quality with outstanding levels of intramuscular fat deposition and redness in meat compared to other commercial pig breeds. Recent studies^13,14^ have revealed diverse genomic regions and genes involved in the superior meat quality of the breed. Although those studies have revealed several candidate genomic regions associated with its phenotype, the understanding of its full genomic architecture is still incomplete.

**Fig. 1:**
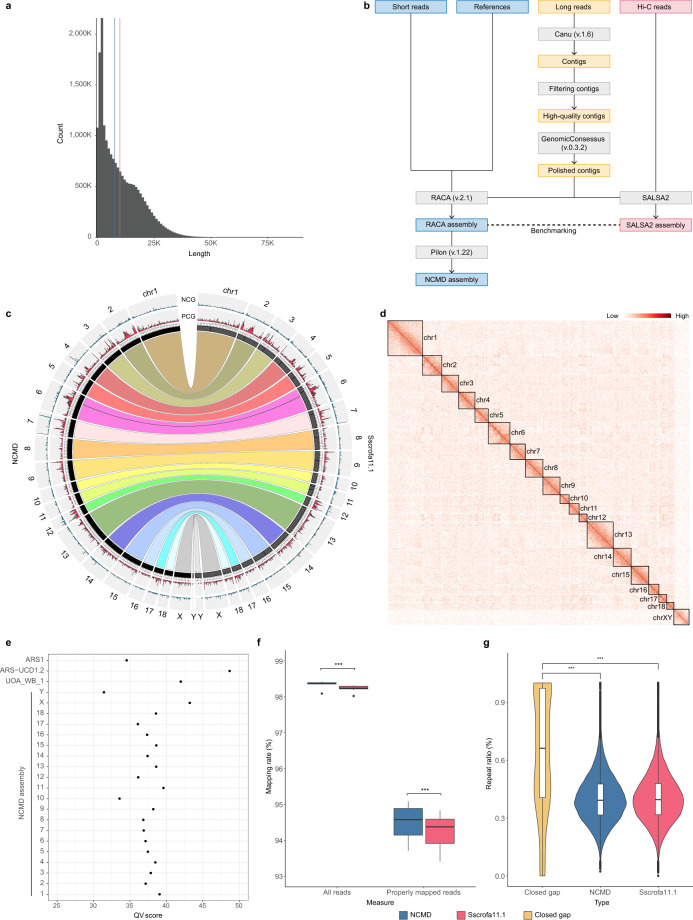
Chromosome-level genome assembly (NCMD) of the Nanchukmacdon pig breed. (**a**) Length distribution of raw Pacbio subreads of Nanchukmacdon. The mean and median subread lengths were 9.90 Kbp (Red line) and 7.57 Kbp (Blue line), respectively. (**b**) Workflow for the genome assembly of Nanchukmacdon. Grey-colored boxes represent programs, and yellow-, blue-, and red-colored boxes indicate the input and output of the programs for building contigs, reference-guided scaffolding and Hi-C based scaffolding. The box named “References” represents the genome sequences of related species. (**c**) Comparison results between NCMD and pig reference genome assembly (Sscrofa11.1). Ribbons show the syntenic relations between the two genomes, and bar plots in the outer rings represent the distribution of protein-coding (PCG, colored by red) and non-coding (NCG, colored by blue) genes in each assembly. (**d**) Heatmap of the Nanchukmacdon Hi-C reads mapped to the NCMD assembly (resolution: 1 Mbp). The Darkness of the red color represents the strength of chromosome interactions. (**e**) QV scores of chromosomes in the NCMD assembly and the genome assemblies of goat (ARS1)^34^, cow (ARS-UCD1.2)^64^ and water buffalo (UOA_WB_1)^65^. (**f**) Comparison of the mapping rates of all reads (“All reads”) and properly mapped reads (“Properly mapped reads”) against the NCMD and pig reference genome assembly (***p < 0.001, Mann-Whitney U test). (**g**) Comparison of repeat ratios in closed gap regions and other genomic regions of the NCMD and pig reference genome assembly (***p < 0.001, Mann-Whitney U test).

Here, we constructed a chromosome-level genome assembly of Nanchukmacdon by the reference-guided approach using only Illumina short reads and PacBio long reads. For genome assembly, we generated short and long reads using the DNA sample of an adult male of Nanchukmacdon (Fig. 1a, Table 1). Then, a genome assembly was constructed using the sequencing reads with the reference-guided approach (Methods, Fig. 1b). The 80.14x raw PacBio subreads were assembled and polished to generate 1,942 high-quality contigs supported by at least 50 PacBio subreads (Fig. 1a). The high-quality contigs were built with N50 of 5.87 Mbp, the GMASS score^15^ of 0.92, and the BUSCO complete score^16^ of 93.4% (Supplementary Table 1).

**Table 1:** Summary of sequencing data of Nanchukmacdon.

Data	Sequencer	Average read length (bp)	Sequencing depth	Insert size
Long reads	PacBio Sequel	9896.56	80.14x	—
Short reads (Paired end)	Illumina HiSeq X Ten	151	193.09x	170 bp
Short reads (Mate-pair)	Illumina HiSeq X Ten	151	242.83x	10–15 Kbp
Hi-C reads	Illumina HiSeq X Ten	151	18.55x	—
RNA-seq reads	Illumina HiSeq X Ten	101	—	180–250 bp

To generate a chromosome-level assembly, the high-quality polished contigs were then further assembled by an improved version of RACA^17^ that can utilize both the genome information of related species and diverse types of sequencing data. Here, we used pig (Sscrofa11.1)^18^ as ingroup, cow (ARS-UCD1.2)^19^ and goat (ARS1)^20^ as outgroup, and short reads of the Nanchukmacdon for scaffolding (Methods). To validate the resulting assembly (hereafter called the RACA assembly), we further generated and used Hi-C sequencing data to build an additional chromosome-level assembly by SALSA2 (hereafter called the SALSA2 assembly; See Technical Validation). On comparing the two assemblies, the RACA assembly was found to be superior to the SALSA2 assembly in terms of both continuity and accuracy.

The RACA assembly was used to build the final assembly after one more polishing step using short reads. The final assembly consisted of 1,077 sequences with a total length of 2.48 Gbp and N50 of 138.61 Mbp. The GMASS and the BUSCO complete scores also increased to 0.99 and 93.5% (Supplementary Table 1). In addition, the top 20 long scaffolds completely corresponded to the regular chromosomes of the pig reference assembly (Sscrofa11.1), and their minimum and maximum lengths were 5.24 Mbp and 275.04 Mbp, respectively (Fig. 1c, Table 2). The top 20 long scaffolds were defined as the NCMD assembly and used for further analyses.

**Table 2:** Statistics of the NCMD and pig reference genome assembly (Sscrofa11.1). The statistics were calculated using only chromosome-level scaffolds of each assembly.

Assembly	NCMD	Sscrofa11.1
No. of sequences^†^	20	20
Total length (Gbp)	2.38	2.44
Min length (Mbp)	5.24	43.55
Max length (Mbp)	275.04	274.33
N50 (Mbp)	138.77	139.51
No. of gaps	855	533
Total gap length (bp)	52,881	29,848,884
BUSCO score (Assembly)	C:93.1%[S:92.5%,D:0.6%],F:3.8%,M:3.1%,n:4104	C:93.7%[S:93.2%,D:0.5%],F:3.5%,M:2.8%,n:4104
No. of protein-coding genes	20,588	21,301
No. of non-coding genes	8,651	8,971
BUSCO score (Protein-coding genes)	C:94.5%[S:49.8%,D:44.7%],F:2.8%,M:2.7%,n:4104	C:99.3%[S:40.8%,D:58.5%],F:0.1%,M:0.6%,n:4104

^†^ The number of chromosome-level scaffolds

As shown in Table 2, genome size, N50, and BUSCO results also showed that the accuracy of the NCMD assembly is comparable to as the pig reference. The GMASS score of the NCMD assembly was 0.99 against pig reference genomes. In the Hi-C heatmap, the 20 chromosome-level scaffolds in the NCMD assembly were clearly distinguished (Fig. 1d). The QV scores of chromosomes in the NCMD assembly ranged from 31.42 (chromosome Y) to 43.20 (chromosome X), which is reasonable compared to the QV scores of the genome assemblies of other species (Fig. 1e). The mappability of the short reads from ten Nanchukmacdon individuals^13^ was also significantly higher for the NCMD assembly than that of the pig reference genome assembly (Fig. 1f).

The NCMD assembly was successfully used to close 143 out of 495 gaps in the pig reference genome assembly (1.21 Mbp in Sscrofa11.1) by identifying 7.34 Mbp of additional non-N bases absent in the pig reference genome assembly (Supplementary Table 2). The closed gap regions contained significantly more repeat sequences than other genomic regions (p < 0.001, Mann-Whitney U test; Fig. 1g). In addition, a total of 34 closed gaps were parts of 627 exons in 57 genes (Supplementary Table 3; refer to the following subsection for gene annotation).

**Fig. 2:**
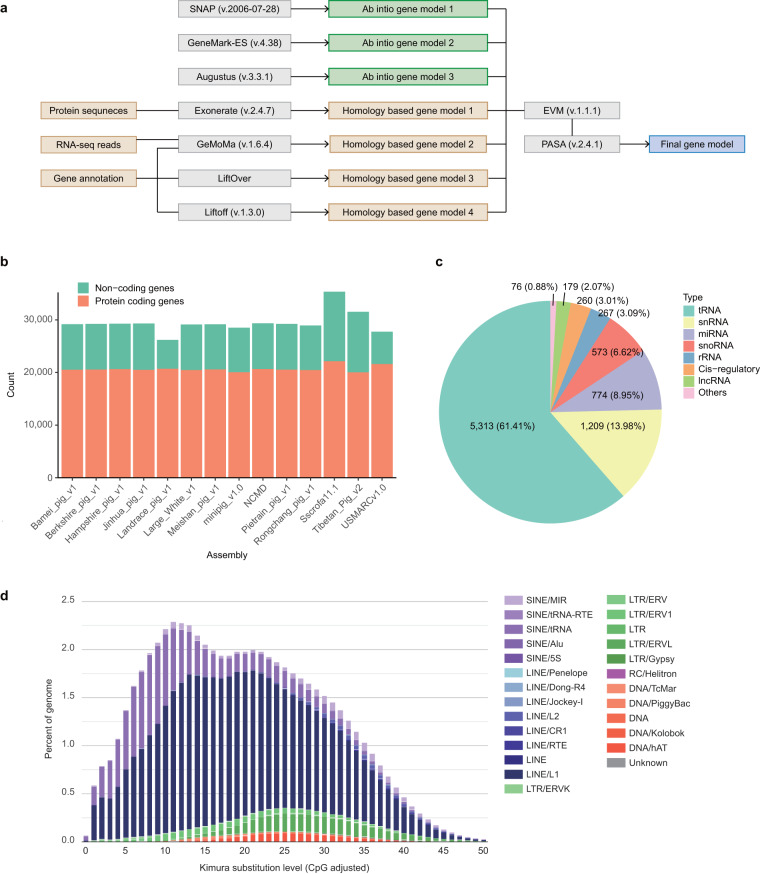
Genome annotation of NCMD assembly. (**a**) Workflow for annotating protein-coding genes. Grey-colored boxes represent programs, and green- and brown-colored boxes respectively indicate their input and output for ab initio and homology-based prediction. The boxes named “Protein sequences” and “RNA-seq reads” represent the protein sequences obtained from the UniProtKB/Swiss-Prot database and RNA-seq data from 24 different tissues of Nanchukmacdon, respectively. “Gene annotation” means the collection of reference gene annotations of related six species (cow, goat, human, pig, mouse and sheep) for GeMoMa program or the gene annotation of pig for LiftOver and Liftoff program. (**b**) Gene annotation statistics for the assemblies of diverse pig breeds. The annotation statistics of 13 pig assemblies except the NCMD assembly were obtained from the Ensembl database (Release 109). (**c**) Statistics for the annotated non-coding genes of the NCMD assembly. (**d**) Sequence divergence of repetitive elements in the NCMD assembly.

For annotating genes, RNA samples were prepared and sequenced from 24 different tissues of the Nanchukmacdon individual which was used for whole genome sequencing (Supplementary Table 4). Using a combination of ab initio and homology-based prediction approaches with the RNA sequencing data, a total of 20,588 protein-coding genes with an average length of 47.06 Kbp were annotated in the NCMD assembly (Table 2, Fig. 2a). The annotated protein-coding genes contained an average of 21.42 exons (average length: 301.96 bp) and transcribed an average of 1.98 transcripts (average length: 3.26 Kbp). Functions were successfully predicted for 17,896 (86.92%) of the annotated protein-coding genes (Methods).

The number of annotated protein-coding genes in the NCMD assembly was highly similar to the ones obtained from other pig breeds (Fig. 2b). The quality of the annotated protein-coding genes was assessed with BUSCO genes. As shown in Table 2, 3,880 (94.5%) out of the 4,104 BUSCO genes were completely annotated in the NCMD assembly. While the gene annotation for the NCMD assembly was built using only short read RNA-seq data, its BUSCO score was comparable with the pig reference annotation which was built using both long read Iso-seq data as well as short read RNA-seq data^21^. In addition, 24 BUSCO genes not present in the annotated genes of the pig reference were found in the annotation of the NCMD assembly (Supplementary Table 5). In the case of non-coding genes, a total of 8,651 non-coding genes were annotated from the NCMD assembly (Fig. 2c).

**Table 3:** The number of matched and mismatched bases in syntenic regions between the NCMD and pig reference genome assembly (Sscrofa11.1).

Chromosome	Match	Mismatch	Match / Total
1	270,633,269	539,424	0.998
2	146,662,781	420,263	0.997
3	130,937,868	425,642	0.997
4	128,427,692	307,617	0.998
5	101,619,062	362,037	0.996
6	167,000,026	484,255	0.997
7	118,773,752	380,777	0.997
8	136,957,235	413,526	0.997
9	136,660,501	463,160	0.997
10	67,560,251	274,356	0.996
11	77,680,673	301,752	0.996
12	59,493,038	243,072	0.996
13	204,720,959	496,915	0.998
14	139,462,466	312,847	0.998
15	138,110,563	310,005	0.998
16	78,160,757	283,293	0.996
17	61,528,660	207,843	0.997
18	55,233,540	141,756	0.997
X	112,703,361	125,226	0.999
Y	6,767,646	1,878,658	0.783
Total	2,339,094,100	8,372,424	0.996

We also annotated repetitive elements in the NCMD assembly (Methods). Overall, 41.86% of the NCMD assembly (996.14 Mbp) were masked as repetitive regions (Supplementary Table 6). Consistent with previous studies^21,22^, long interspersed nuclear elements (LINEs) were the most abundant, accounting for 24.75% of the NCMD assembly, followed by short interspersed nuclear elements (SINEs, 9.08%), long terminal repeats (LTRs, 4.66%), DNA transposons (1.94%), and other repeats. Also, LINE L1s and tRNA-derived SINEs showed the most obvious ancient expansion and recent inactivation (Fig. 2d). This high-quality NCMD assembly and annotation provide foundational resources for the genomic analyses of pig and related species.

## Methods

### DNA and RNA sequencing

DNA was extracted from the blood sample of a male Nanchukmacdon (10 weeks old) using procedures verified and approved by the National Institute of Animal Science in the Republic of Korea in compliance with the ARRIVE guidelines^23^. For long read sequencing, DNA libraries were constructed using 20 Kbp SMRTbell Template Prep Kits and sequenced by the PacBio Sequel instrument. For short read sequencing, DNA libraries were constructed using the TruSeq DNA Library Prep kit HT and the Nextera Mate Pair Sample Prep Kit with a gel-plus protocol for paired-end and mate-pair libraries, respectively. Insert sizes for the paired-end and mate-pair libraries were 170 bp and 10–15 Kbp, respectively. The libraries were sequenced by the Illumina HiSeq X Ten with a read length of 151 bp. For generating Hi-C reads, DNA libraries were constructed using the Truseq Nano DNA library Kit and sequenced by the Illumina HiSeq X Ten with a read length of 151 bp (Table 1).

Using a TRIzol extraction reagent, total RNAs were extracted from 24 different tissues (appendix, backfat, blood, rib, skull, bone marrow, brain, colon, heart, groin, kidney, liver, lung, foreleg, hindleg, nipple, phren, sirloin, skin, small intestine, spleen, stomach, tenderloin, and testicle) of the same individual from which the DNA was extracted. The extracted RNAs were subjected to TruSeq stranded mRNA Sample Prep Kit and sequenced with a read length of 101 bp by the Illumina HiSeq X Ten sequencer (Table 1, Supplementary Table 4).

### Genome assembly

Figure 1b shows the workflow of the genome assembly. Contigs were generated by Canu (v.1.6)^24^ using raw PacBio subreads with default parameters except for genomeSize = 2455 m, which was estimated from the pig reference genome assembly (Sscrofa11.1). The contigs not supported by at least 50 subreads were filtered out and not used in subsequent assembly steps. The remaining contigs were polished using GenomicConsensus (v.2.3.3; https://github.com/PacificBiosciences/GenomicConsensus) with –algorithm = best parameter by mapping all subreads to the contigs using pbalign (v.0.3.2; https://github.com/PacificBiosciences/pbalign).

Two different approaches were adopted to build a chromosome-level genome assembly from the contigs: (i) reference genome-assisted assembly and (ii) Hi-C read-based assembly. For the reference genome-assisted assembly, the contigs were further assembled using the improved version of RACA^17^ (manuscript in preparation) that can assemble given contigs (or scaffolds) using both genome sequences of multiple related species and various types of read sequences of a target species. For the preparation of input data for RACA, the quality of short reads was controlled by the IlluQC module in NGSQCToolkit (v.2.3.3)^25^ with N A parameters. The high-quality short reads were then mapped to the contigs using BWA MEM (v.0.7.17-r1198)^26^ with default parameters. Regarding reference species, pig was used as an ingroup species, and cow and goat were used as outgroup species. Their assemblies were obtained from the NCBI database^27^ (Sscrofa11.1^18^, ARS-UCD1.2^19^, and ARS1^20^ for pig, cow, and goat, respectively). Repeat sequences in each assembly were masked using RepeatMasker (v.4.0.5)^28^. Pairwise whole-genome alignments between the pig genome assembly against each of the other ones were constructed using LASTZ (v.1.04.00)^29^ with the following parameters: E = 150 H = 2000 K = 4500 L = 2200 M = 254 O = 600 Q = human_chimp.v2.q T = 2 Y = 15000. By using the mapping files of short reads and the whole-genome alignment files as input, RACA was run with 150 Kbp as the minimum size of synteny blocks and the other parameters are described in Supplementary Table 7. The divergence time among species was obtained from TimeTree^30^, and the time between the pig reference and Nanchukmacdon was set to an arbitrarily small number (1 in this study) because they belong to the same species. Potential genome structure-level misassemblies in the chosen assembly were manually corrected based on syntenic relationships with the pig reference genome, which were generated by the improved version of RACA. Finally, correction of other misassemblies and closing of gaps using short reads were performed twice using BWA MEM (v.0.7.17-r1198) and Pilon (v.1.22)^31^ with default parameters to produce the final chromosome-level assembly of Nanchukmacdon. The chromosome names of the NCMD assembly were decided based on the syntenic relationships with the pig reference genome.

For the Hi-C read-based assembly, the quality control of raw Hi-C reads was performed by using the same approach used for the short reads. The cleaned Hi-C reads were mapped to the contigs of Nanchukmacdon using BWA MEM (v.0.7.17-r1198) with default parameters. The mapping results were filtered by Arima filtering protocol (https://github.com/ArimaGenomics/mapping_pipeline). Using the filtered mapping results and the contigs as input, SALSA2^32^ was run with the following parameters: -m yes -e DpnII -i 10.

### Benchmarking scaffolding approaches

The two assemblies generated by the two different scaffolding approaches were compared in terms of assembly contiguity and completeness using N50, BUSCO^16^ and GMASS^15^ scores as described in the following subsection. The reference genome-assisted assembly (hereafter called the NCMD assembly) was chosen for further analysis. We also calculated the number of contigs connected with each other during scaffolding and the physical coverages at assembly gaps in each assembly using in-house Perl scripts. The physical coverages were calculated with the mate-pair reads used to construct the NCMD assembly. Pairwise whole genome alignment between the two assemblies was conducted using MUMmer4^33^.

### Assembly quality assessment

Assembly completeness was assessed by BUSCO (v.3.0.2) using the mammalia_odb9 dataset. Genome structural similarity between the pig reference genome assembly (Sscrofa11.1) and the NCMD assembly was measured by GMASS with five resolutions (100, 200, 300, 400, and 500 Kbp) for synteny block recreation. In addition, synteny blocks generated with 300 Kbp resolution were used to calculate the synteny coverage against each assembly, which was defined as the total length of genomic regions belonging to all synteny blocks divided by the genome size.

Whole-genome sequence alignments between the two assemblies were used to count the number of matched and mismatched bases. The quality value (QV) score representing the estimated per-base accuracy of a genome assembly was also calculated as described in previous studies^34^ by mapping the short reads of Nanchukmacdon against the NCMD assembly. In addition, the short reads of ten additional Nanchukmacdon individuals obtained from a previous study^13^ were also aligned to the NCMD assembly using BWA MEM (v.0.7.17-r1198) with default parameters to assess the read mappability. Of note, the short reads that were used to construct the NCMD assembly were excluded in this assessment for improving the reliability of the measure. The mappability-related statistics, such as the number of mapped reads, were calculated using BAMtools (v.2.5.1)^35^.

### Closing gaps in the pig reference genome assembly

Gaps in the pig reference genome assembly (Sscrofa11.1) were closed using the approach described in a previous study^36^. Gap positions in the pig reference genome assembly were obtained from the NCBI database. For each gap, two 10 Kbp flanking sequences from each side of the gap were extracted and aligned to the NCMD assembly using NUCmer in the MUMmer package (v.4.0.0beta2)^33^ with -f -r -l 15 -c 25 parameters. Gaps were considered as closed when the following criteria were met: (i) two flanking sequences were aligned and the alignment length of each sequence was larger than 5 Kbp, (ii) the average percentage identity of alignments for two flanking sequences was higher than 90%, (iii) the orientation and order of the alignments for two flanking sequences were the same in the pig reference genome and the NCMD assembly, and (iv) there were no Ns in the aligned sequences of the NCMD assembly against the two flanking sequences.

To compare the ratio of repetitive sequences between the closed gaps and the other regions, the repeats in the pig reference genome and NCMD assembly were first masked using RepeatMasker (v.4.0.5) with a pig repeat library. All sequences in the two assemblies were then divided into non-overlapping windows of length 50 Kbp. The repeat ratios calculated in each of the windows of the closed gaps and the other regions were compared using the Mann-Whitney U test conducted by the wilcox.test function in the R package (https://www.r-project.org). The distribution of the repeat ratios in the whole assembly was plotted using the ggpubr package (https://github.com/kassambara/ggpubr). In addition, the existence of the annotated Nanchukmacdon genes (described in the following subsection) in the closed gap regions was examined to identify any related functions.

### Gene annotation

Using sequenced RNA data, protein-coding genes in the NCMD assembly were first annotated by a combined method of *ab initio* and homology-based prediction. For the *ab initio* prediction, SNAP (v.2006-07-28)^37^, GeneMark-ES (v.4.38)^38^ and Augustus (v.3.3.1)^39^ were run for the repeat-masked NCMD assembly. For SNAP and Augustus, which are based on the hidden Markov model (HMM), a novel HMM for pig genes was produced using gene annotation information of the pig reference assembly (Sscrofa11.1)^18^ downloaded from the NCBI database.

For the homology-based prediction, three different approaches, (i) Exonerate^40^, (ii) GeMoMa^41^, and (iii) LiftOver^42^ and Liftoff^43^ were used. For Exonerate (v.2.4.7), protein sequences were downloaded from the UniProtKB/Swiss-Prot database (release-2020_04)^44^, and mapped against the NCMD assembly using Exonerate with --maxintron 50000 parameter. The value 50000 is the maximum size of introns inferred based on the gene annotation information of the pig reference assembly. GeMoMa (v1.6.4) was run using the mapping data of Nanchukmacdon RNA-seq reads for the NCMD assembly with gene annotation information of assemblies of six species, i.e., cow (ARS-UCD1.2)^19^, goat (ARS1)^20^, human (GRCh38.p13)^45^, pig (Sscrofa11.1)^18^, mouse (GRCm38.p6)^46^, and sheep (Oar_rambouillet_v1.0)^47^, downloaded from the NCBI database. The RNA-seq reads of Nanchukmacdon were mapped to the NCMD assembly using STAR (v.2.7.1a)^48^. For each gene annotation, DNA sequences of coding exons were extracted from the matched assembly and translated to peptide sequences using the GeMoMa Extractor module. The peptide sequences were then aligned to the NCMD assembly using TBLASTN (v.2.9.0)^49^. The pig gene annotation information was also converted into the NCMD assembly by using LiftOver and Liftoff (v.1.3.0). To reduce false positives from conversion results, only genes whose locations in each assembly were consistent with the syntenic relationships between the two assemblies were retained.

All predicted gene models by the *ab initio* and homology-based approaches were integrated into the final gene annotation using the PASA annotation pipeline (v.2.4.1)^50^ and Evidence Modeler (v.1.1.1)^51^. Functions of genes in the final gene annotation were predicted by aligning the protein sequences of the predicted genes to the protein sequences of the pig reference assembly using BLASTP (v.2.9.0)^49^ with a percentage identity cutoff of 50% and an e-value cutoff of 0.01. For genes whose functions were not found by BLASTP, their functions were inferred from the corresponding genes in the pig reference assembly which were found using the conversion results of LiftOver and Liftoff.

Non-coding genes for diverse types of RNAs, including rRNA, snRNA, and miRNA, were annotated by using the Rfam database^52^ and Infernal (v.1.1.3)^53^ with -Z 4969.1164 --cut_ga --rfam --nohmmonly parameters. The tRNAscan-SE (v.2.0.5)^54^ and RNAmmer (v.1.2)^55^ were used to annotate non-coding genes for tRNA and rRNA, respectively. The three annotation results were merged into a non-redundant non-coding gene annotation using an in-house Perl script.

The quality of the final gene annotation of the pig reference and NCMD assembly was compared by BUSCO (v.3.0.2) with the mammalia_odb9 database based on their protein sequences. To calculate the gene density in chromosomes, the pig reference and NCMD assembly were divided into 1 Mbp-sized bins, and the number of genes per type observed in each bin was counted.

### Repeat annotation

A *de novo* repeat library was first constructed using RepeatModeler (v.2.0.1.)^56^, and unknown repeats were additionally classified by BLAST (v.2.9.0)^49^. The *de novo* repeat library was next merged with the library of pig taxon-specific repeats extracted from the repeat database of RepeatMasker^28^ using RepeatMasker utility (queryRepeatDatabase.pl). Finally, repetitive elements in the NCMD assembly were annotated by RepeatMasker (v.4.0.5) with default parameters and the constructed repeat library. Utility scripts provided by RepeatMasker (calcDivergenceFromAlign.pl and createRepeatLandscape.pl) were used to draw repeat landscapes.

### Collinearity comparison of the chromosome-level assemblies of pig breeds

A whole-genome sequence alignment of the NCMD assembly against the pig reference genome assembly (Sscrofa11.1) was constructed using LASTZ (v.1.04.00)^29^. Synteny blocks between them were then constructed by the synteny block detection program in InferCars^57^ with a resolution of 300 Kbp. The Hi-C reads of Nanchukmacdon were also aligned to the NCMD assembly, and the Hi-C contact map was constructed using the HiC-Pro pipeline (v.2.11.4)^58^ with default parameters and 1 Mbp size of bin. The Hi-C contact maps were visualized using HiCPlotter (v.0.8.1)^59^. The syntenic relationships and breakpoint regions between the two assemblies were visualized using Circos^60^.

## Data Records

The NCMD assembly has been deposited at DDBJ/ENA/GenBank under the accession JAVFKH000000000^61^. The gene annotation has been deposited at FigShare^62^. Raw whole genome Pacbio long read, Illumina short read, Hi-C sequencing data and Illumina RNA-seq data of 24 tissues are available at NCBI SRA under the project of PRJNA967127^63^.

## Technical Validation

### Genome assembly

The quality of NCMD assembly was identified in terms of assembly contiguity and completeness. The N50, QV, BUSCO and GMASS score were respectively 138.77 Mbp, 93.1%, 37.48, and 0.99, which is comparable to the pig reference genome (Table 2). We also calculated the ratio of matched and mismatched bases in the synteny blocks. The 99.64% of bases in the NCMD assembly were exactly matched with those in the pig reference genome assembly (Table 3). The mappability of the short reads against the NCMD and pig reference genome assembly was also compared using the public short reads of ten additional Nanchukmacdon individuals. Mapping rates of both all reads and properly mapped reads against the NCMD assembly were significantly higher than those against the pig reference genome assembly for all ten samples (p < 0.001, Mann-Whitney U test; Fig. 1f).

### Scaffolding approach

To evaluate the performance of the reference genome-assisted scaffolding approach used for creating the NCMD assembly, an additional assembly was generated by scaffolding high-quality contigs using a Hi-C read-based scaffolding tool SALSA2^32^ (Methods; Fig. 1b). For this purpose, Hi-C reads with medium depth (18.55x) were also generated (Table 1). Using the Hi-C reads, the high-quality contigs generated using long reads were scaffolded and the resulting assembly, hereinafter referred to as the SALSA2 assembly, was compared with the previous version of the final NCMD assembly, hereinafter referred to as the RACA assembly, which was the one before applying the last polishing step with short reads to ensure a fair comparison.

The RACA assembly was superior to the SALSA2 assembly in terms of both continuity and accuracy (Supplementary Table 1). For the continuity, the RACA assembly showed an even lower number of sequences (1,077) and larger N50 (138.69 Mbp) than the SALSA2 assembly (1,408 and 14.15 Mbp respectively). The maximum length of scaffolds in the RACA assembly was 275.17 Mbp, which is similar to the size of chromosome 1 in the pig reference assembly, while the maximum length was just 91.46 Mbp in the SALSA2 assembly. With respect to the accuracy, the RACA assembly showed a higher GMASS score against the pig reference assembly (0.99) and BUSCO score (93.6%) than the SALSA2 assembly (0.95 and 93.5% respectively).

**Fig. 3:**
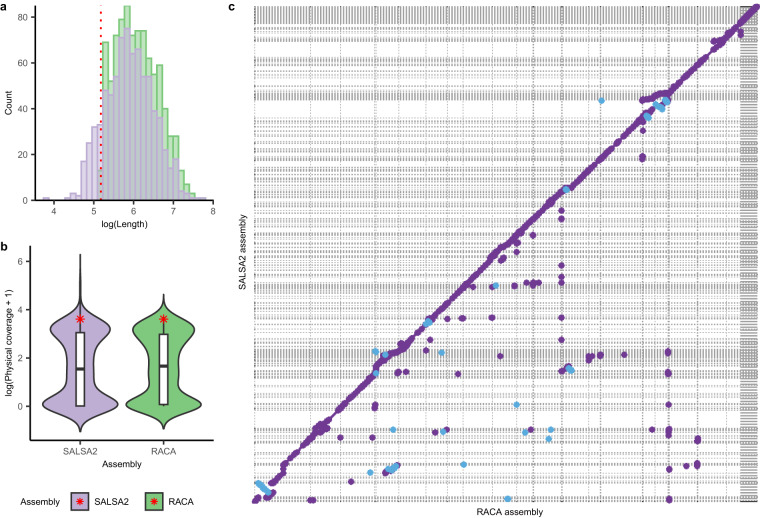
Comparison results between the reference-guided assembly and Hi-C read-based assembly represented by RACA and SALSA2 respectively. (**a**) Distributions of lengths of contigs which were connected with others when creating scaffolds. A red dotted line denotes 150 Kbp which is the minimum length of a synteny block used as a resolution parameter in the RACA program. (**b**) Distributions of physical coverages at assembly gaps in each assembly. The physical coverages were calculated with mate-pair reads used for constructing the NCMD assembly. Red asterisk marks represent genome-wide averages of the physical coverage in each assembly. (**c**) A dot plot of pairwise whole-genome alignment between the two assemblies. Purple and blue dots represent local forward and reverse alignments between them, respectively.

We also compared length distributions of contigs that were connected to others when creating scaffolds (Fig. 3a). While the distribution of physical coverage in assembly gaps between contigs was similar between the two approaches as shown in Fig. 3b,the RACA assembly was constructed by connecting more contigs with all lengths larger than the resolution value used for assembly (150 K). Especially, both assemblies showed bimodally distributed physical coverages in assembly gap regions with the modes at higher coverage located close to the genome-wide average of physical coverage (the red star in Fig. 3b). Furthermore, the pairwise comparison of these two assemblies also revealed that the SALSA2 assembly is highly fragmented compared with the RACA assembly, despite a high correspondence between them (Fig. 3c).

### Gene annotation

The annotation quality of NCMD assembly was compared to that of pig reference by BUSCO (v.3.0.2) with mammalia_odb9 database based on their protein sequences. As shown in Tables 2, 94.5% of the BUSCO genes were completely annotated in the NCMD assembly. In addition, 24 BUSCO genes that are not present in the annotated genes of the pig reference were found in the annotation of the NCMD assembly (Supplementary Table 5).

We also compared the gene density in chromosomes for the pig reference (Sscrofa11.1) and the NCMD assembly. Genomic distributions of both protein-coding and non-coding genes were similar between the NCMD and pig reference genome assembly (Fig. 1c) confirming the completeness and accuracy of the annotated genes from the NCMD assembly.

### Supplementary information

Supplementary Tables

## Data Availability

Codes for benchmarking scaffolding approaches and constructing a non-redundant non-coding gene annotation are freely available at GitHub (https://github.com/jkimlab/NCMD_study). The other programs used in this study were executed following their manuals. The version and used parameters of programs are specified in the Methods section.
